# Rasch-Built Overall Disability Scale for Chronic Idiopathic Axonal Polyneuropathy

**DOI:** 10.1212/WNL.0000000000218266

**Published:** 2026-07-17

**Authors:** Gerjan M. van der Star, Catharina G. Faber, Pieter A. van Doorn, Nicolette C. Notermans, Alexander F.J.E. Vrancken, Ingemar S.J. Merkies

**Affiliations:** 1 Department of Neurology and Neurosurgery, UMC Utrecht Brain Center, University Medical Center Utrecht, the Netherlands;; 2 Department of Neurology, Maastricht University Medical Center+, the Netherlands;; 3 Department of Neurology, Erasmus University Medical Center, Rotterdam, the Netherlands; and; 4 Department of Neurology, Curacao Medical Center, Willemstad.

## Abstract

**Background and Objectives:**

Chronic idiopathic axonal polyneuropathy (CIAP) is a common type of chronic polyneuropathy that often affects health-related quality of life. Currently, no patient-reported disease-specific outcome measure is available to assess functional deficit in patients with CIAP. The aim of this study was to construct a patient-reported Rasch-built interval scale for patients with CIAP.

**Methods:**

A historic prospective cohort study was conducted to develop a CIAP-specific Rasch-built overall disability scale (CIAP-RODS). The preliminary scale (pre-CIAP-RODS) comprised 196 items, including 146 activity and participation items selected from the World Health Organization International Classification of Functioning, Disability and Health and 50 expert-derived items formulated in collaboration with a CIAP patients advocacy group. Participants were patients with CIAP who were requested to score their perceived difficulty to perform each item as (0) unable to perform; (1) able to perform, but with difficulty; or (2) easily performed, without difficulty. For test-retest reliability studies, 150 participants completed the pre-CIAP-RODS twice with an interval of 2–4 weeks. The pre-CIAP-RODS was subjected to Rasch analyses (RUMM2030+) to develop the final CIAP-RODS, and external validity was assessed by examining associations with the European Quality of Life 5 Dimensions 3 Level Version (EQ-5D-3L).

**Results:**

Of 551 eligible patients invited, 268 completed the pre-CIAP-RODS (mean age 72 years; 31% female; median disease duration 14 years). Patients with relevant comorbidities or newly identified risk factors of polyneuropathy were excluded. The pre-CIAP-RODS was subjected to Rasch analyses and did not meet the Rasch model expectations. In a systematic and stepwise manner, items were removed based on disordered thresholds, misfit statistics, local dependency, previously reported patient perceptions, and clinical applicability, resulting in a final 24-item CIAP-RODS that fulfilled Rasch model requirements. Test-retest reliability was good, internal validity was robust (person separation index 0.95), and significant associations between CIAP-RODS person location and EQ-5D-3L item scores indicated good discriminative external validity.

**Discussion:**

The 24-item CIAP-RODS is a disease-specific interval measure developed for detection of activity and participation limitations in patients with CIAP. Use of the CIAP-RODS in future studies is recommended to evaluate longitudinal changes in the disease course. Further studies are needed to determine responsiveness and cross-cultural validity.

## Introduction

Chronic idiopathic axonal polyneuropathy (CIAP) is the second most commonly diagnosed type of polyneuropathy after diabetic polyneuropathy, affecting approximately one-third of patients with chronic polyneuropathy.^1^ Symptoms follow a length-dependent pattern, initially affecting the feet, after which the lower legs and eventually the hands may become involved over the course of years.^2-7^ Through sensory or sensorimotor symptoms (e.g., tingling, numbness, pain, and mild distal weakness), CIAP can result in walking difficulties that sometimes require the use of assistive devices but patients remain ambulatory.^5-8^ It may also interfere with various activities of daily living, including leisure, social participation, and work.^9,10^ Collectively, these factors can contribute to a significant reduction in health-related quality of life.^10^

Currently, no patient-reported disease-specific outcome measures are available for patients with CIAP to estimate disease impact and evaluate changes in the disease course. Despite the current lack of treatment, there is a need for patient-centered outcome measures. With an expected 25% increase in the prevalence of chronic axonal polyneuropathy over the next 20 years due to aging of the population, an outcome measure that adequately captures the associated burden of disease is essential.^4^ The aim of this study was to present the development and evaluation of a Rasch-built overall disability scale for CIAP (CIAP-RODS) and to examine its scientific soundness.^11,12^

## Methods

### Standard Protocol Approvals, Registrations, and Patient Consents

All participants signed written informed consent before participating in the study. The study protocol was approved by the local medical research ethics committee of the University Medical Center Utrecht (UMCU).

### Participants

For questionnaire development using Rasch analysis, a minimum number of 250 participants are preferred.^13^ We approached 551 patients with CIAP between December 2022 and April 2023. These patients were all diagnosed with CIAP in a standardized manner at the UMCU in the Netherlands between March 1993 and July 2022 and were identified based on their participation in earlier studies.^14-17^ The Neuromuscular Clinic of the UMCU is an accredited tertiary referral center with longstanding dedicated expertise in polyneuropathies, and CIAP in particular. The diagnosis of CIAP was made using predefined criteria: symptoms and signs consistent with polyneuropathy, history taking and medical history revealing no identifiable risk factors of polyneuropathy, first onset of symptoms after age 40, no family history of polyneuropathy, comprehensive laboratory testing to exclude alternative etiologies, and nerve conduction studies to rule out a demyelinating polyneuropathy and confirming large nerve fiber involvement.^14^

Patients were not eligible for participation if they had developed new risk factors of polyneuropathy since their previous hospital visit. In addition, patients with a history or new diagnosis of other neurologic comorbidities (e.g., spinal stenosis, mononeuropathy, cerebrovascular disease, and Parkinson disease), or with other non-neurologic diagnoses resulting in temporary disability (e.g., ankle/hip fracture, failed knee replacement, plantar fasciitis, arthritis, infection, postoperative recovery, and cardiac failure) were excluded. This was assessed through review of the electronic medical record and a screening questionnaire covering newly developed risk factors and medical history, including recent neurologic and non-neurologic diagnoses. Participant characteristics were obtained from the electronical medical records.

### Questionnaire Development

The CIAP-RODS questionnaire was developed through the application of previously published standardized requirements for scale development.^12,13,18^ A 196-item prephase CIAP-Rasch-built overall disability scale (pre-CIAP-RODS) was composed of a list of 146 activity and participation items selected from the World Health Organization International Classification of Functioning, Disability and Health and an additional 50 items based on input of experts in the field in collaboration with patients living with CIAP represented in the CIAP patients advocacy group at the patient organization for neuromuscular diseases in the Netherlands (Spierziekten Nederland). The aim of this collaboration was to refine items (components of existing items, more difficult variants of existing items, and new items).^19^ Participants were requested to score their perceived difficulty to perform items as (0) unable to perform; (1) able to perform, but with difficulty; or (2) easily performed, without difficulty.

### Additional Outcome Measures

The European Quality of Life 5 Dimensions 3 Level Version (EQ-5D-3L), a health-related quality-of-life questionnaire, was completed once by all participants to assess external validity of the final CIAP-RODS. The EQ-5D-3L consists of 5 domains (mobility, self-care, usual activities, pain/discomfort, and depression/anxiety) for which participants score whether they have (0) no problems, (1) some problems, or (2) a lot of problems.^20^ The EQ-5D-3L also includes a visual analogue scale (VAS) to assess the participants' self-rated health state (range 0–100, from worst to best imaginable health state).

### Assessment Procedure

Participants received the pre-CIAP-RODS and EQ-5D-3L, accompanied with standardized written instructions by post. Participants were instructed that there are no right and wrong answers, but to base their answer on how they feel. They were instructed to fill in “able to perform, but with difficulty” if performing an activity would be possible with the use of a device (e.g., walking stick) or other forms of assistance (e.g., family/partner), and to fill in “unable to perform” if it would be impossible to perform despite the use of a device or assistance. For every item and in case of doubt, the participants were instructed to answer to the best of their personal judgment and as close as possible to their ability to complete the activity. When the ability of a participant varied throughout the day or week, the participant was requested to choose the answer that best represented the activity as performed most of the time. Items can be interpreted according to the patient's usual pattern, which may vary between patients. The patient instruction manual is available on request. The researcher (G.M.v.d.S.) was available for questions of participants by telephone and email. The pre-CIAP-RODS questionnaire was completed twice in a subset of participants with an interval of 2–4 weeks for test-retest reliability studies. Participants were given the option to complete the second form digitally.

### Rasch Analyses

We used Rasch methodology to construct an interval measure, in contrast with ordinal scales based on classical test theory with associated disadvantages due to the lack of a fixed unit on the scale's range.^21^ The pre-CIAP-RODS was subjected to Rasch Unidimensional Measurement Model (RUMM2030+ software) to determine whether it is consistent with Rasch model requirements.^22,23^ The Rasch methodology has been previously described in numerous educational articles, which provide an accessible and comprehensive overview.^24-26^ The Partial Credit Model was set as the default. Stepwise analyses were conducted to construct a final CIAP-RODS with the aim of fulfilling all Rasch requirements, defined as a logit (log odds) scale with sufficient fit statistical parameters that is unidimensional, without disordered thresholds, item bias, or local dependency.^24,25,27^

The following person factors were taken into account for the scale's construction, to assess for differential item functioning (DIF): age (<65, 65–74, ≥75 years), sex (female vs male), and disease duration (<10, 10–14, 15–19, ≥20 years). Disease duration was defined as the interval between onset of first symptoms and study inclusion. For age and disease duration, the class intervals contain an approximately equal distribution of participants.

Unidimensionality was monitored throughout the analyses and assessed using principal component analysis and the independent *t* test, as described previously.^28,29^ Using principal component analysis, 2 different Rasch analyses were performed based on the 2 most divergent sets of items (6 items per set) and the person ability estimates of both Rasch analyses were compared. As both subsets should render the same estimate of person ability for every participant, a proportion of significant *t* tests for all individual participants below 0.05 (5%), or overlap of the lower 95% CI with 0.05 (5%), indicates unidimensionality.

### Clinical Applicability

Considering the large number of items, we chose to further select items based on clinical applicability. Drawing on reported patient perceptions of a previously published Rasch-built Overall Disability Scale, items with a poor patient-reported understanding and uncertainty, and items similar to those reported, were omitted by retrospective assessment of face validity.^30^ In addition, items that were not applicable to all participants, for example, requiring the use of specific objects a patient may not possess (e.g., bathtub and garden tools), were removed to improve generalizability.

### Reliability and Validity Studies

Internal validity of the final CIAP-RODS was determined by assessment of the Person Separation Index (PSI). For clinical proper discriminatory ability, the PSI is preferably higher than 0.9.^27^ Test-retest reliability studies were performed to determine consistency of item hierarchy (logits) and participant ability (logits) over time. This was assessed through graph analyses using 95th CIs and linear regression studies (expressed as *R*^2^).^31^ For reliability studies, a minimum sample size of 80 patients was required. To ensure a more stable assessment, we applied a minimum sample size of 150 patients.^32^

Because there are no specific functional metrics available to assess functional deficits in patients with CIAP, we chose not to validate the CIAP-RODS against a same-level outcome measure, but directly against a patient-reported health-related quality-of-life measurement. We aimed to capture the impact of the CIAP-RODS on an internationally widely used general quality-of-life scale, such as the EuroQol. External validity of the final CIAP-RODS was, therefore, determined through correlation studies with the EQ-5D-3L VAS score. Moreover, we examined whether CIAP-RODS person location estimates differed significantly across response categories for individual EQ-5D-3L items, which was visualized using boxplots. We evaluated all 5 EQ-5D-3L domains, although the domains of mobility, self-care, and usual activities were the most conceptually aligned with the items of the CIAP-RODS. Further analyses were performed using R (version 4.2.2 (2022-10-31)) for MacOS and Stata 13.0 for Windows. *p* Values lower than 0.05 were considered statistically significant. Bonferroni correction for multiple testing was applied to assessment of item fit statistics in step 2 and to the assessment of differential item functioning across all analyses. We used a threshold of 0.05/n, where n is the total number of items tested in the analysis, which varied as the items were sequentially removed.^33^

### Data Availability

Anonymized data are available on request from any qualified investigator.

## Results

### Participants and Data Quality Control

**Figure 1:**
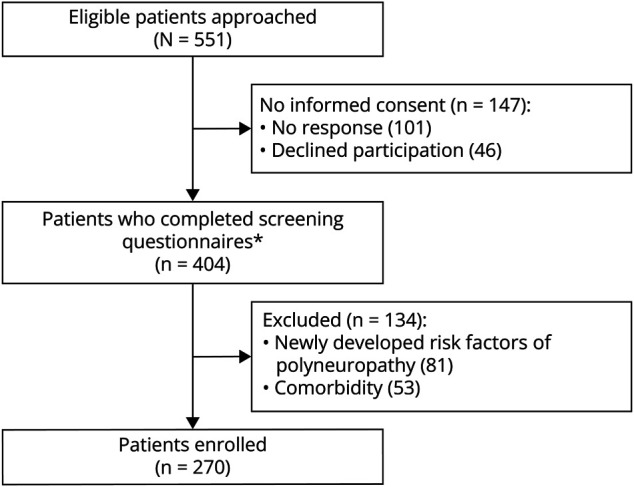
Flowchart of Patient Inclusion *The screening questionnaires included assessment of newly developed risk factors of polyneuropathy since last hospital visit and medical history, including recent neurologic and non-neurologic diagnoses.

Patient inclusion is depicted in Figure 1. A total of 270 participants completed the pre-CIAP-RODS. Two participant records were omitted because of missing data, defined as >10% missing values on the pre-CIAP-RODS, leaving 268 participants' records available for further investigation. Using this cutoff, no items had to be omitted because of missing data. The mean (SD; range) age was 72 (8.5; 48–97) years, and the cohort consisted of 84 (31%) female and 184 (69%) male participants. The median (25th–75th percentiles; range) disease duration was 14 (9–17; 2–45) years. A total of 150 participants completed the pre-CIAP-RODS twice, after 2–4 weeks, for evaluation of reliability without having access to the first assessment data.

### Initial Rasch Analyses on the Pre-CIAP-RODS

The 196-item pre-CIAP-RODS did not perform in accordance with the Rasch model expectations. The fit residuals deviated substantially (mean item fit residuals −0.333, SD 1,094; mean person fit residuals −0.342, SD 1,509), and a significant χ^2^ for item-trait interaction and analysis of variance (ANOVA) fit were not consistent with the Rasch model requirements. Multidimensionality was present, indicated by a proportion of 0.091 (95% CI 0.060–0.127) of the *t* tests that were significant.

### Data Handling of the Pre-CIAP-RODS to Fit Rasch Modeling

#### Step 1

Class intervals were continuously monitored throughout the following steps. Three items showed disordered thresholds and were subsequently removed. Two items were removed in which only 2 of the 3 answer categories were used by participants (191 items remaining).

#### Step 2

The individual item fit statistics of 15 items demonstrated misfit (misfit statistics and/or fit residuals exceeding ±2.5) and were removed stepwise (176 items remaining). No participant records were removed.

#### Step 3

A large number of correlations were present between item residuals. All item pairs with a correlation above 0.20 were evaluated starting with the highest correlations (>0.7, >0.6, … up to >0.20).^34^ For each item set, the items were examined on item location, DIF, and overdiscrimination or underdiscrimination on the category probability curve. The item with the least favorable characteristics was removed. Eventually, a total of 134 items were removed in a stepwise manner (42 items remaining).

#### Step 4

Further adjustments were needed to meet Rasch model expectations. Therefore, we lowered the *p* value for the fit residuals to 0.01, resulting in the removal of 3 additional items (39 items remaining). Summary statistics improved (items' mean fit residuals −0.356, SD 0.701, and persons' mean fit residuals −0.263, SD 0.883, χ^2^ item-trait interaction: *p* = 0.27, ANOVA fit statistic: *p* = 0.06).

#### Step 5

Owing to overlap in content of items “open/close a door” and “open a door with a key,” we chose to omit “open/close a door” because this item was less clearly defined and less favorable for Rasch expectations (38 items remaining).

#### Step 6

To further reduce the number of items, 8 items were removed that were comparable to items with previously reported poor understanding and uncertainty (e.g., “go to a hospital”).^30^ In addition, 3 items were removed that referred to activities not reasonably applicable to all participants (“step in/out of bath”) to enhance generalizability. Adjustment was needed to meet Rasch model expectations, which was achieved by removing 3 items with the most aberrant misfit statistics (24 items remaining).

#### Step 7

One item showed uniform DIF (walk on toes) on sex. Female participants experienced “walk on toes” as more difficult. Because the location and associated location thresholds of this item were necessary to maintain and even item location distribution, this item was not omitted but split for male and female participants. Because examination of unidimensionality is not available in RUMM2030+ software after having split items for DIF, we examined this before splitting the items. Two subsets of items were created (6 most positively loaded and 6 most negatively loaded) based on principal components analyses. The proportion of significant *t* tests between the 2 groups of items was 0.080 (95% CI 0.049–0.112), indicating unidimensionality.

An overview of the original 196-item list, including which items were removed at which steps, is given in eTable 1.

**Figure 2:**
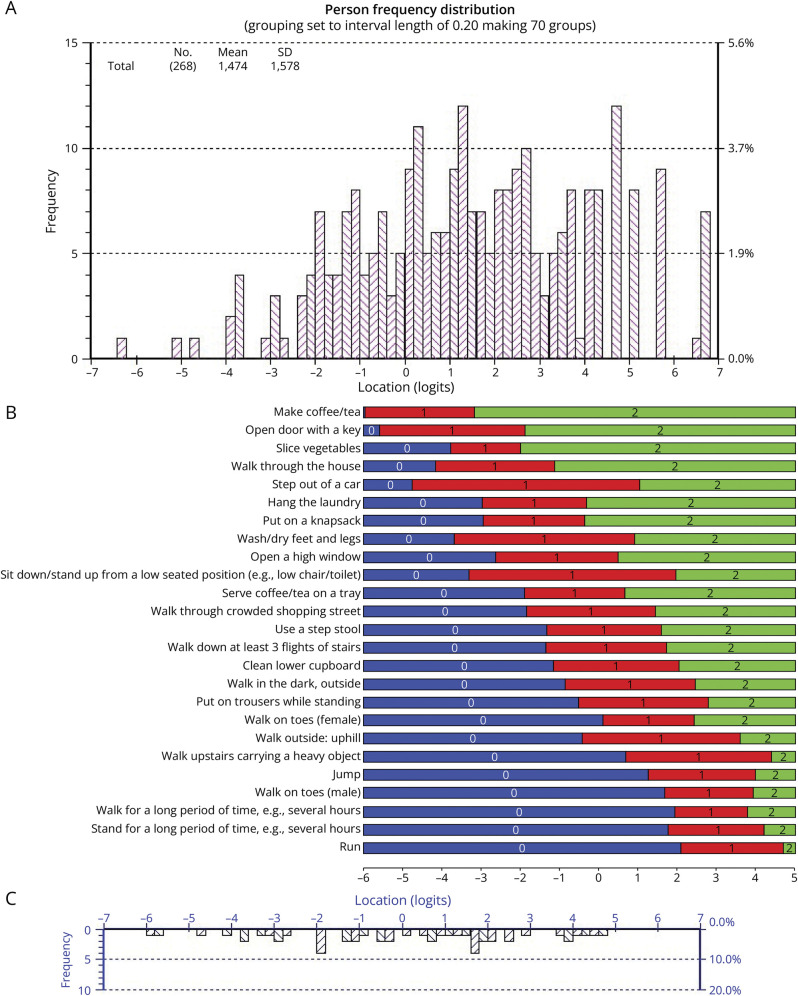
Final 24-Item CIAP-RODS (A) Distribution of activity and participation assessment (person ability) of 268 patients on CIAP-RODS. (B) Threshold map of the 24 items (25 inquiries) as part of the final CIAP-RODS. The expected response for each item related to the patients' ability is depicted. The easiest item was “make coffee/tea,” and the most difficult item was “run.” Because zero logits was set as the average of item difficulty and patients' ability, a patient with a mean score would be able to walk through a crowded shopping street (this item requires −0.188 logits) easily and would likely have a higher chance of being able to perform the easier tasks (having a lower logit location score). Conversely, this patient will have a greater difficulty to perform more difficult tasks and will most probably fail on these. (C) Distribution of the location of item thresholds of final 24 items (24 items, 3 responses per item, meaning 2 thresholds per item). CIAP-RODS = Rasch-built overall disability scale for chronic idiopathic axonal polyneuropathy.

Finally, we succeeded in constructing the CIAP-RODS. This final CIAP-RODS contains 25 inquiries originating from 24 remaining items (Figure 2). It sufficiently meets the Rasch model expectations (item fit residuals: mean −0.379, SD 0.732; person fit residuals: mean −0.248, SD 0.769; item-trait χ^2^: *p* = 0.25, ANOVA fit statistic: *p* = 0.10, degrees of freedom = 75), except for 1 significant residual correlation still being present. The easiest item for participants was “make coffee/tea,” while the most difficult item was “run.” Item difficulty ranged from −4.563 to 3.430 logits, and participant location ranged from −6.400 to 6.657. One of the 268 participants (0.4%) showed a floor effect, and 8 participants (3.0%) showed a ceiling effect on the final model (Figure 2).

### Validity and Reliability

**Figure 3:**
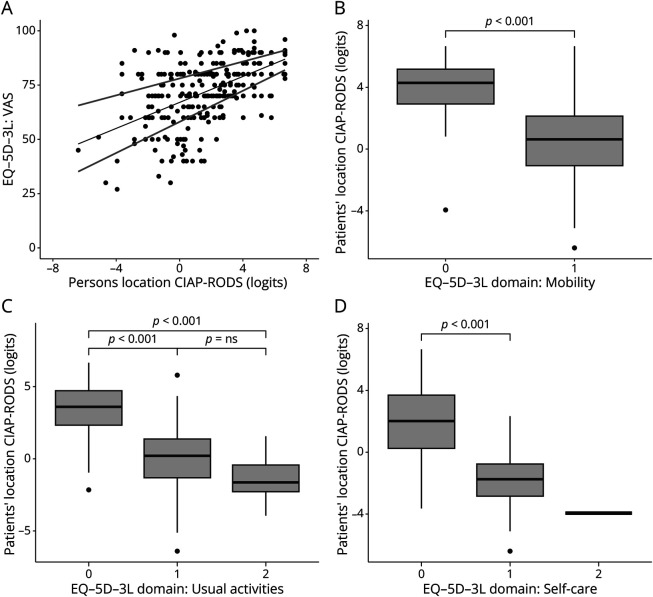
Association Between CIAP-RODS Person Location and EQ-5D-3L VAS Score for Patients' Self-Rated Health State With Fitted Quantile Regression Lines at 25th, 50th, and 75th Percentiles (A), and Box Plots for Association Between CIAP-RODS Person Location and Individual EQ-5D-3L Item Scores for the Domains of Mobility (B), Usual Activities (C), and Self-Care (D) The functioning of patients on EQ-5D-3L domains is scored as (0) no problems, (1) some problems, or (2) a lot of problems. A higher patients' location on the CIAP-RODS indicates a lower amount of disability. CIAP-RODS = Rasch-built overall disability scale for chronic idiopathic axonal polyneuropathy; EQ-5D-3L = European Quality of Life 5 Dimensions 3 Level Version; VAS = visual analogue scale.

**Figure 4:**
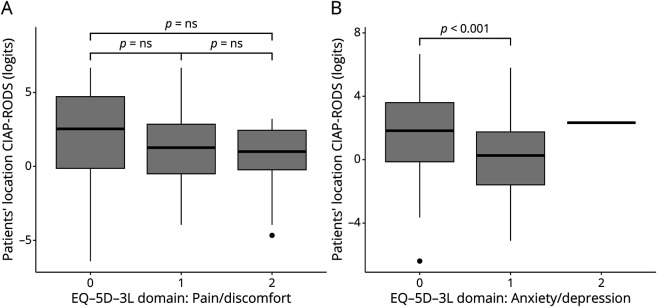
Box Plots for Association Between CIAP-RODS Person Location and Individual EQ-5D-3L Item Scores for the Domains of Pain/Discomfort (A) and Anxiety/Depression (B) The functioning of patients on EQ-5D-3L domains is scored as (0) no problems, (1) some problems, or (2) a lot of problems. A higher patients' location on the CIAP-RODS indicates a lower amount of disability. CIAP-RODS = Rasch-built overall disability scale for chronic idiopathic axonal polyneuropathy; EQ-5D-3L = European Quality of Life 5 Dimensions 3 Level Version.

**Figure 5:**
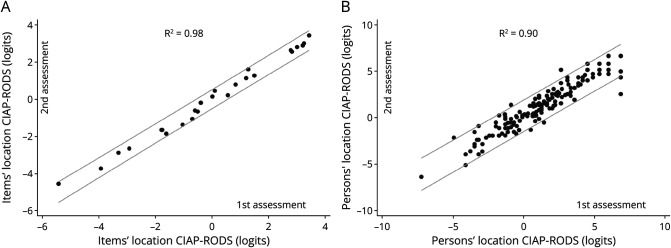
Hierarchy of Item Difficulty (A) and Person Location (B) of CIAP-RODS in First vs Second Assessment The light gray lines represent the 95% CI. Almost all items and patients were located within the 95% CI, reflecting ideal reliability. CIAP-RODS = Rasch-built overall disability scale for chronic idiopathic axonal polyneuropathy.

There was a moderate correlation between the final CIAP-RODS person estimates and EQ-5D-3L VAS score (Spearman ρ = 0.46, *p* < 0.001) (Figure 3A). For all EQ-5D-3L domains, except for “pain/discomfort,” the CIAP-RODS person estimates differed significantly between EQ-5D-3L answer options, indicating good discriminative validity (Figure 3, B–D). The domains of pain/discomfort and anxiety/depression, which are less conceptually aligned with the CIAP-RODS, are presented in Figure 4, A and B. The internal reliability was robust (PSI 0.95). There was optimal test-retest reliability for item difficulty and participant ability (Figure 5, A and B).

## Discussion

In this article, we present the development of a patient-reported Rasch-built activity and participation interval scale tailored specifically to measure disease impact in patients with CIAP. Excellent discriminatory validity and reliability was demonstrated for the final CIAP-ROD`S.

Several patient-reported outcome measures have been developed for specific axonal polyneuropathies (diabetic, HIV-related, chemotherapy-induced, familial amyloid neuropathy).^35^ Except for a RODS for patients with chemotherapy-induced peripheral neuropathy (CIPN-RODS) and a RODS for patients with familial amyloid polyneuropathy, all other outcome measures for these axonal polyneuropathies function at the ordinal level. In the field of immune-mediated polyneuropathies, the use of Rasch-built patient-reported outcome measures is already well established.^18,36-38^ For patients with CIAP, no disease-specific patient-reported outcome measures are available. With aging of the population and growing prevalence of CIAP, it is increasingly important to quantify the impact of CIAP at the individual patient level and to assess the broader burden of disease at the population level.^5^

The modified Rankin score, Medical Research Council (MRC) sum score, change in the proportion of patients using walking aids, and change in the proportion of patients with pain or other positive sensory symptoms are commonly used as ordinal outcome measures although there is no consensus on which of these is best.^35,39,40^ However, these outcome measures are not developed specifically for polyneuropathy or CIAP, and most focus on a single component of disability (e.g., muscle weakness as measured by the MRC sum score and presence of pain).

Because the CIAP-RODS assesses impact of disease using participation and activity items, it inherently captures all relevant components of disability, rather than addressing a specific component of disability. Key strengths of the CIAP-RODS are that it permits to measure disability at an interval-level scale, allowing for parametric statistical analysis, and that it is disease-specific for patients with CIAP.^26^ Through external validation, we demonstrated that the CIAP-RODS score captures multiple dimensions of health-related quality of life, in addition to assessing disability in daily living. In addition, through patient participation, the clarity and comprehension of the items were improved. Finally, the study population was considered representative as patients were referred to the UMCU from across the Netherlands, from both rural and urban areas and across a broad range of disease severity.

One of the limitations is that 1 item pair as part of the final questionnaire was accepted having residual correlations exceeding the 0.2 cutoff. We chose not to omit one of these 2 items because these were necessary for a more even distribution of item location and corresponding thresholds. Because removing one of these items did not result in a substantial change in model fit determinants, we believe that the impact of the correlation is minor. Despite these residual correlations, the Rasch model retains excellent performance and fulfills Rasch model expectations. Second, although patient perception is key to develop relevant patient-reported outcome measures, patient-perceived relevance of items was not formally evaluated. Incorporating patient perspectives at an early stage, for example, during the construction phase, may have further improved the relevance of the items of the final questionnaire and could be considered in future development of Rasch questionnaires. Third, because CIAP predominantly affects older individuals, comorbidity may influence disability as well as quality of life. Although exclusion criteria were applied to limit the impact of temporary comorbidity, we did not assess chronic comorbidity in sufficient detail to allow comparison of its prevalence with that of the general population. In future research, it may be valuable to include comorbidity as a person factor in Rasch analysis, to allow assessment of its influence on specific items through DIF.

Although the CIAP-RODS is developed specifically for patients with CIAP, it might also have a broader application to these other forms of chronic acquired axonal polyneuropathies with a typical distal symmetric sensory predominant phenotype (e.g., diabetes, chronic kidney disease, thyroid disease, vitamin deficiencies, excessive alcohol consumption, and chemotherapy). However, because some manifestations are disease-specific, for example, foot ulcers in diabetic polyneuropathy due to concurrent peripheral vascular disease, this needs to be studied further. Furthermore, when comparing the CIAP-RODS with the CIPN-RODS, a RODS for patients with CIPN, both scales generally identify items related to walking in challenging contexts (e.g., walking for longer periods of time, walking the stairs, and running) as the most difficult tasks while items pertaining to disability of the upper extremity are identified as less difficult. Although few of the items are identical, the order of item difficulty appears different, presumably reflecting a different spectrum of disability. This may be attributed to higher levels of pain experienced by patients with CIPN, as well as the broader impact of the underlying malignancy and its associated treatments on daily functioning, disability, and overall quality of life.

Because this study has only been performed in Dutch patients with CIAP, future research will have to assess the cross-cultural validity of this scale. The slow disease progression and lack of available treatments did not permit assessment of short-term responsiveness in our study. Longitudinal studies are necessary to evaluate responsiveness.

In conclusion, the 24-item CIAP-RODS is a disease-specific interval measure that enables measurement of restrictions in activity and participation in patients with CIAP with excellent validity and reliability.

## Glossary

ANOVA: analysis of variance
CIAP: chronic idiopathic axonal polyneuropathy
CIAP-RODS: Rasch-built overall disability scale for patients with CIAP
CIPN-RODS: Rasch-built overall disability scale for patients with chemotherapy-induced peripheral neuropathy
EQ-5D-3L: European Quality of Life 5 Dimensions 3 Level Version
DIF: differential item functioning
MRC: Medical Research Council
PSI: Person Separation Index
RUMM: Rasch Unidimensional Measurement Model
UMCU: University Medical Center Utrecht
VAS: visual analogue scale
